# A comprehensive analysis of antibiotic resistance genes in the giant panda gut

**DOI:** 10.1002/imt2.171

**Published:** 2024-02-06

**Authors:** Feilong Deng, Yanhua Han, Yushan Huang, Desheng Li, Jianmin Chai, Linhua Deng, Ming Wei, Kai Wu, HuaBin Zhao, Guan Yang, Jiangchao Zhao, Ying Li, Chengdong Wang

**Affiliations:** ^1^ Guangdong Provincial Key Laboratory of Animal Molecular Design and Precise Breeding, College of Life Science and Engineering Foshan University Foshan China; ^2^ Department of Animal Science, College of Life Science and Engineering Foshan University Foshan China; ^3^ National Conservation and Research Centre for Giant Pandas/China Conservation and Research Centre for the Giant Panda Chengdu China; ^4^ Department of Ecology, College of Life Sciences Wuhan University Wuhan China; ^5^ Department of Infectious Diseases and Public Health City University of Hong Kong Kowloon, Hong Kong, SAR China; ^6^ Department of Animal Science, Division of Agriculture University of Arkansas Fayetteville Arkansas USA

## Abstract

In this study, we have successfully constructed a comprehensive database of metagenome‐assembled genomes (MAGs) pertaining to the gut microbiota of the giant panda. Through our analysis, we have identified significant reservoirs of antibiotic resistance genes (ARGs), namely *Escherichia coli, Citrobacter portucalensis*, and *Klebsiella pneumoniae*. Furthermore, we have elucidated the primary contributors to ARGs, including *Streptococcus alactolyticus* and *Clostridium SGBP116*, in both captive and wild pandas. Additionally, our findings have demonstrated a higher prevalence of ARGs in the metagenome, with notable expression of the *RPOB2* gene in *S. alactolyticus*. Crucially, 1217 ARGs shared homology with human gut ARGs, underscoring the interaction relationship between pandas and human microbiomes. These findings are instrumental in understanding the antibiotic resistance landscape in the giant panda's gut, providing a framework for developing strategies to combat antibiotic resistance and safeguard the health of this endangered species.

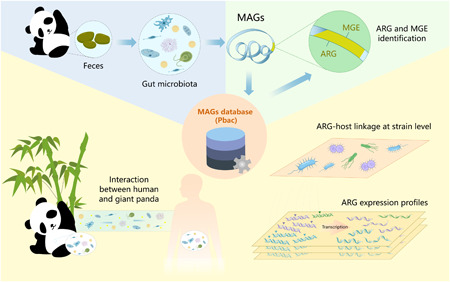

The giant panda is a symbolically important and endangered species, drawing international attention to its conservation. Although substantial initiatives by the Chinese government and the scientific community have led to an encouraging rise in its population, giant pandas still face significant survival hurdles. These vulnerabilities arise from challenges such as habitat fragmentation, which hinders genetic exchange, suboptimal reproduction rates due to diverse reasons, and susceptibility to diseases from various pathogens [1].

The complex microbial communities within the giant panda's gut play a vital role in their health, digestion, and immune functions [2]. Due to the pandas' unique dietary habits (mainly bamboo), the gut microbiota likely plays a critical role in digesting indigestible fibers and maximizing protein utilization under limited protein availability [3]. Concurrently, pathogenic microbes within the gut may pose a challenge to protecting giant pandas. Notably, gastrointestinal diseases are more prevalent in captive pandas compared with other systemic diseases, with frequent antibiotic use leading to increased antibiotic resistance [4]. In addition, a heightened concern within the realm of conservation biology is the potential transmission of antibiotic‐resistant bacteria from anthropogenic sources to the giant panda [5]. Such transmission may precipitate the emergence of antibiotic resistance genes (ARGs) within the species, exacerbating health risks for this already endangered population [6, 7]. The presence of these ARGs may undermine the effectiveness of therapeutics used to treat giant pandas and other species that could acquire these resistance genes [8].

Understanding the distribution of ARGs within the gut microbiota of giant pandas, as well as identifying the microbial hosts, is pivotal for formulating effective strategies to curtail antibiotic resistance. Contemporary literature underscores the multifaceted influences on the ARGs present in the gut microbiome, with variables such as age, dietary patterns, seasonal variations, habitat characteristics, geographical location, and management practices being of particular importance [5, 7, 9, 10, 11, 12]. Although previous research employing metagenomic sequencing has predominantly focused on the distribution and key drivers affecting ARGs in giant pandas, there remains considerable scope to delve deeper. In light of this, the present study aims to (1) develop a comprehensive metagenome‐assembled genomes (MAGs) database specific to the giant panda gut microbiota, facilitating an in‐depth exploration of ARG profiles and highlighting strain level differentials; (2) utilize meta‐transcriptomic sequencing techniques to elucidate the active expression patterns of ARGs in captive giant pandas; and (3) investigate the potential for horizontal gene transfer, specifically focusing on the transfer of ARGs from the human gut microbiota to that of giant pandas, illuminating possible anthropogenic impacts on this endangered species.

## RESULTS AND DISCUSSION

### A comprehensive database of MAGs for the giant panda

In our previous study [3], we reconstructed 610 medium‐quality MAGs with a coassembly strategy. Here, we revisited these samples using an independent assembly strategy, which has been demonstrated to yield genomes of superior quality [13]. Merging the newly assembled MAGs with our prior results, we obtained 2684 MAGs meeting the quality threshold (completeness ≥ 50%, contamination＜10%, length ≥ 500 kb). Impressively, 960 (35.77%) of these MAGs surpassed the high‐quality benchmark (completeness ≥ 90%, contamination＜5%). We applied a *de novo* cluster threshold at 99% similarity, which was considered a strain‐level [14], resulting in 1193 nonredundant MAGs that included 354 high‐quality MAGs. Compared with our previous study and a recently published study [15], more nonredundant MAGs (1193 *vs*. 408 and 820) and high‐quality MAGs (354 *vs*. 149 and 174) were included in this study, even though more sequencing data were used in the previous study [15].

**Figure 1:**
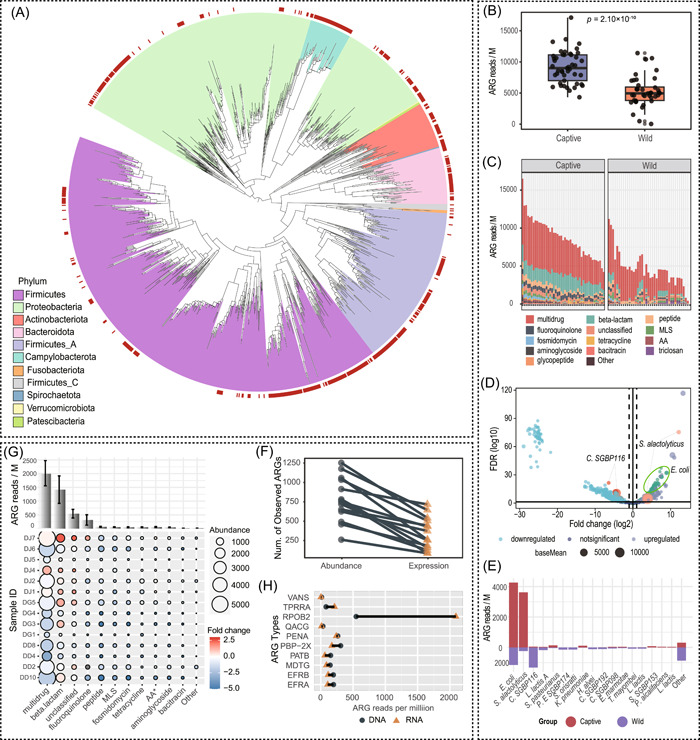
Phylogenetic relationships of metagenome‐assembled genomes (MAGs), and comparative analysis of antibiotic resistance gene (ARG) profiles in the gut microbiota of giant pandas. (A) Phylogenetic tree illustrating the relationships of MAGs derived from the gut of giant pandas. Each background color corresponds to a different bacterial phylum. The outer circle is color‐coded, with red branches indicating novel species. (B) Relative ARG abundance in the gut microbiota of captive versus wild giant pandas. Statistical significance was assessed using analysis of variance (ANOVA). (C) Stacked bar chart representing ARG classes in the gut microbiota of both panda populations. “AA” denotes the ARG class of aminoglycoside:aminocoumarin. (D) Volcano plot comparing relative ARG abundance to ARG expression levels. Highlighted circles/points indicate ARGs hosted by specific bacterial species. (E) Comparative contributions of average ARG abundance from various host species in both wild and captive giant pandas. (F) Comparison of detected ARG types in both metagenomic (observed ARGs) and meta‐transcriptomic (expressed ARGs) datasets. Only ARG reads with counts exceeding 1 per million were included in the analysis. Corresponding samples from individual giant pandas are connected with lines. (G) Visualization of metagenomic abundance versus meta‐transcriptomic expression levels for different ARG classes in captive pandas’ samples. The diameter of each circle corresponds to the metagenomic abundance of its ARG class. The color gradient, shifting between blue and red, indicates the log2‐transformed fold change in meta‐transcriptomic expression relative to metagenomic abundance for each ARG class. The bar plot positioned above displays the cumulative abundance of expressed ARGs. AA represents the ARG class of aminoglycoside:aminocoumarin. (H) Depiction of the mean relative abundance and expression rates for specific ARGs that are associated with *Streptococcus alactolyticus* in the giant panda gut. The ARG type of TPRRA represents the type of TRUNCATED_PUTATIVE_RESPONSE_REGULATOR_ARLR.

From the 1193 nonredundant MAGs, 71 bacterial families were identified across 11 phyla (Figure 1A). Firmicutes dominated the MAGs taxonomy (503 MAGs or 42.16%), succeeded by Proteobacteria (357 MAGs or 29.92%), Firmicutes A (167 MAGs or 14.00%), and Bacteroidota (59 MAGs or 4.95%). At the genus level, 1193 MAGs corresponded to 171 existing genera in genome taxonomy database (GTDB), while 11 MAGs lacked GTDB references, clustering at 85% similarity into six genus clusters. At the species level, 672 MAGs were mapped to 219 existing species. The remaining 521 MAGs, absent from the GTDB, clustered into 228 clusters at 95% similarity.

Intriguingly, *Turicibacter SGBP131*, containing 23 representative MAGs, has not been annotated as a known species, suggesting its potential as a novel species. Additionally, 39 novel candidate species were identified, each containing a minimum of three strains. These include *Campylobacter D SGBP104* (25 MAGs), *Turicibacter SGBP131* (23 MAGs), and *Clostridium SGBP116* (20 MAGs). Such findings suggest a considerable abundance of previously uncharacterized species in the gut microbiome of the giant panda. These results serve as a comprehensive reference point for subsequent analyses. For accessibility, all MAGs and related information have been made available at our online database: http://www.pbac.top.

Of note, a primary limitation of our database is the disproportionate representation of captive versus wild giant pandas, which may have constrained the genomic diversity of the gut microbiome. Furthermore, we observed inconsistencies between the phylogenetic tree and annotations at the phylum level (Figure 1A), a discrepancy also noted in the study by Miranda et al. [16], suggesting that the validity of the classification warrants further deliberation.

### Identifying key ARG and MGE hosts using the MAGs

Despite the recent progress on ARG diversity, there is still limited information about the ARG hosts. We predicted 10,224 ARGs from representative MAGs (Supporting Information S1: Figure S1A) and found *Escherichia coli* was the predominant host, harboring 65 distinct ARG types, followed by *Citrobacter portucalensis* and *Klebsiella pneumonia*. Furthermore, two species from the *Escherichia* genus, namely *Escherichia marmotae* and *Escherichia sp001660175*, manifested a notably augmented mean gene density, with 27 and 28 ARGs per 2000 genes, respectively (Supporting Information S1: Figure S1B).

We identified 713 mobile genetic elements (MGEs) derived from 233 MAGs. Among these MGEs, 507 were characterized as “*tnpA*.” MAGs identified as *Acinetobacter nosocomialis, Enterococcus B faecium*, and *JGM124 SGBP173* harbored 29, 20, and 19 MGEs, respectively (Supporting Information S1: Figure S1C). The ARG and MGE were defined as co‐occurring if they were located on the same MAG within a 5000‐bp distance [17]. A total of 26 ARGs co‐occurred with MGEs (e.g., *int2, IS91*, and *qacEdelta*), including *CATA, MEXE*, and DNA‐binding protein H‐NS (Supporting Information S1: Table S1). Of these, eight MGE and ARG co‐occurrences were observed on the MAGs of *Escherichia coli*, consistent with Hu et al. [10]. Seven co‐occurrences were associated with three species belonging to the *Acinetobacter* genus. Of note, four co‐occurrences were mediated by *tnpA* present on the MAGs of *Limosilactobacillus mucosae* and *Lactobacillus johnsonii*, which are usually considered probiotics. In a network analysis by Jin et al. [7], *Streptomyces, Acinetobacter, Escherichia*, and *Klebsiella* were significantly associated with ARGs and MGEs. Here, we confirm the predominant ARG and MGE carriers at the species level.

### Comparison of ARGs between wild and captive giant pandas

We next examined the differences in abundance, types, and ARG hosts between captive and wild pandas. The relative abundance of ARGs from captive giant pandas was higher than that of wild ones (*p* = 2.10 × 10^−10^, Figure 1B), aligning with findings from previous research [12]. Regarding ARG classes between wild and captive giant pandas, multidrug was the most abundant ARG type for both, followed by the class of beta‐lactam, peptide, and fluoroquinolone. All the ARG types introduced above were enriched in captive giant pandas (*p* < 0.01) (Figure 1C).

Differential analysis of ARGs revealed that 444 genes were significantly enriched in captive, whereas 347 genes showed significantly higher relative abundance in wild giant pandas (Figure 1D). In captive giant pandas, ARGs that were notably enriched were predominantly associated with *E. coli*, followed by *Streptococcus alactolyticus* (Figure 1E). These two bacteria constituted approximately 88.90 ± 12.13% of the total ARG reads. Conversely, in wild giant pandas, *Clostridium SGBP116* (a novel species) and *E. coli* surfaced as the principal ARG hosts. The potential effects of *Clostridium SGBP116*, which was recognized as a novel species in this study, on the health of wild giant pandas warrant further investigation and consideration.

### Expression patterns of ARGs in captive giant pandas

We next examined the expression patterns of ARGs by comparing the observed with the expressed ARGs. The number of observed ARGs in the metagenomic data set considerably exceeded that of the expressed ARGs in the meta‐transcriptomic data set (*p* = 2.98 × 10^−6^, Figure 1F), implying that certain ARGs remain transcriptionally silent. The most abundant ARG class was the multidrug class, with an average relative abundance of 3154.11 reads/million. Concurrently, the multidrug class was also the most expressed, averaging an abundance of 2017.05 reads/million (Figure 1G). It is also worth noting that the “beta‐lactam” ARG class ranked second in expression (1419.42 reads/million), despite its lower relative abundance, with an average of 770.64 reads/million, indicating a heightened expression activity.

Intriguingly, *RPOB2* (known for resistance to rifampicin) manifested exceptionally high expression, accounting for 67.47% of the total expressed ARGs (Figure 1H). Rifampicin, a commonly utilized drug in humans, has been shown to have activity against several bacterial species in giant pandas, including *Klebsiella pneumoniae* [8]. The potential selective pressures exerted by *RPOB2*‐associated antibiotics, such as rifampicin, warrant further studies, especially concerning the gut microbiome of captive giant pandas.

### The potential impacts of human activities on the gut microbiome of giant pandas

A total of 383,943 ARGs were identified from 13,042 microbial genomes of the human gut, and assigned into 6977 clusters (99%), 1217 of which displayed high genetic similarity (> 99%) to genes found in the gut microbiome of giant panda. These ARGs were associated with 72 distinct species. Compared with wild giant pandas, there was a higher relative abundance of ARGs shared with humans in captive giant pandas (42.37% *vs*. 22.14%). These results suggested that the close genetic resemblance between the ARGs found in giant pandas, particularly those in captivity, and humans underscore a significant interspecies microbial exchange, which may be facilitated by shared environments and close human–animal interactions. Future research should aim to isolate the bacteria‐harboring these ARGs and explore the possible pathways for horizontal gene transfer between humans and giant pandas.

Despite the breakthroughs achieved in our study, it is imperative to recognize the intrinsic limitations inherent in methodologies, including the limitations of algorithm bias and database integrity. Currently, these constraints remain unaddressed due to the lack of more sophisticated alternatives. Thus, it is essential for future research to develop and adopt methodologies with enhanced precision for the quantification of resistance gene expression.

## CONCLUSION

This study has successfully established a MAGs database for the giant panda, encompassing 1193 genomes across 445 species. Our comprehensive analysis of these MAGs has yielded an unprecedented resolution in identifying the hosts of ARGs, as well as their expression profiles. Notably, the homology between many ARGs found in pandas and those present in the human gut microbiome suggests a potential for horizontal gene transfer of ARGs. This research not only provides important bacterial reference genomes for the giant panda but also advances our understanding of ARG dynamics within the giant panda's gut microbiota.

## AUTHOR CONTRIBUTIONS

Feilong Deng, Yanhua Han, and Ying Li drafted the manuscript; Feilong Deng performed analyses with contributions from Yushan Huang, Desheng Li, Jianmin Chai, Ming Wei, and Linhua Deng; Feilong Deng, Kai Wu, and Huabin Zhao provided data visualizations; Guan Yang, Jiangchao Zhao, and Ying Li contributed to critically revising the manuscript; Chengdong Wang, Jiangchao Zhao, and Ying Li contributed to the study concept and design. All authors read and approved the final manuscript.

## CONFLICT OF INTEREST STATEMENT

The authors declare no conflict of interest.

## ETHICS STATEMENT

The ethics application (No. FOSU2020002) was approved by the Animal Care and Use Committee of Foshan University.

## Supporting information


**Figure S1**: Predominant hosts of ARGs and MGEs at the species and strain levels.

**Table S1**: Co‐occurrence of ARGs and MGEs on the MAGs.

## Data Availability

The sequence data utilized in this study can be accessed through the NCBI SRA database using the accession number PRJNA872265. All assembled sequences of MAGs generated in this study were uploaded to the Pbac database (www.pbac.top). Supplementary materials (Materials and Methods, figure, table, graphical abstract, slides, videos and Chinese translated version) may be found in the online DOI or iMeta Science http://www.imeta.science/.
