# Supplementary material for: A comprehensive analysis of antibiotic resistance genes in the giant panda gut
Source: Imeta. 2024 Feb 6;3(1):e171. doi: 10.1002/imt2.171 (PMC10989137; doi:10.1002/imt2.171)
Supplement: Supplementary file 1 — Figure S1: Predominant hosts of ARGs and MGEs at the species and strain levels. [file IMT2-3-e171-s001.docx]

Supporting information to: A comprehensive analysis of ARGs in the Giant Panda gut

Running title: ARGs in the Giant Panda gut

Feilong Deng^1,2#^, Yanhua Han^1,2#^, Yushan Huang^1,2#^, Desheng Li^3#^, Jianmin Chai^1,2^, Linhua Deng^3^, Ming Wei^3^, Kai Wu^3^, HuaBin Zhao^4^, Guan Yang^5^, Jiangchao Zhao^6*^, Ying Li^1,2*^ and Chengdong Wang^3*^

^1^ Guangdong Provincial Key Laboratory of Animal Molecular Design and Precise Breeding, College of Life Science and Engineering, Foshan University, Foshan, China

^2^ College of Life Science and Engineering, Foshan University, Foshan, China

^3^ China Conservation and Research Center of Giant Panda, Key Laboratory of SFGA on Conservation Biology of Rare Animals in The Giant Panda National Park (CCRCGP), Chengdu, China

^4^ College of Life Sciences, Wuhan University, Wuhan, China

^5^ Department of Infectious Diseases and Public Health, City University of Hong Kong, Kowloon, Hong Kong SAR 999077, China.

^6^ Department of Animal Science, Division of Agriculture, University of Arkansas, AR, Fayetteville, USA

^#^Feilong Deng, Yanhua Han, Yushan Huang and Desheng Li contributed equally to this work.

^*^To whom correspondence should be addressed:

Prof**. Jiangchao Zhao** ([jzhao77@uark.edu](mailto:jzhao77@uark.edu)), Prof**. Ying Li** ([yingli@fosu.edu.cn](mailto:yingli@fosu.edu.cn)), and Prof**.** **Chengdong Wang** ([wolongpanda@qq.com](mailto:wolongpanda@qq.com))

**Materials and Methods**

**1. MAGs construction**

A total number of 322 fecal samples from captive giant pandas and wild giant pandas were involved in this study, which were described with details in our previous publication [1]. In addition, 7 metagenomic sequencing samples of wild giant pandas were collected [2]. The raw reads were pre-processed using the Kneaddata pipeline v0.7.2 (<https://bitbucket.org/biobakery/kneaddata>). In summary, Trimmomatic v0.39 [3] trimmed raw reads for low-quality sections (Phred < 20 over 4 bp) and discarded reads under 60 bp post-trimming. Bowtie2 (version 2.2.5) [4] was used to identify and eliminate reads of giant panda (GCF_002007445.1) and bamboo genomes (GCA_017311315.1 and GCA_011038535.1). We re-analyzed these samples using the independent assembly strategy using Spades (v3.15.5) software [5] with “—meta” mode and default parameters to minimize contamination and inter-sample interference. This method enhances the acquisition of high-quality MAGs, according to Olm et al. [6]. Next, the assembled scaffolds longer than 2000 bp were subjected to genome binning using MetaBAT2 (version 2.12.1) [7] with default parameters. The dRep software (v3.4.3) [6] was used to remove redundant bins with the parameter ‘-sa 0.99’. We used CheckM v1.1.3 (lineage_wf) [8] to determine the final quality of bins (MAGs), including completeness and contamination, and only kept MAGs with length > 500kb, completeness ≥ 50%, and contamination < 10%. The tRNA and rRNA genes of MAGs were annotated using Barrnap v0.9 (https://github.com/tseemann/barrnap) and tRNAscan-SE (v 2.0.9) [9], respectively. The taxonomy assignment of these MAGs was inferred using GTDB-TK v1.5.0 [10] with the GTDB reference (R06-RS202, March 21, 2022) [11]. MAGs that cannot be assigned to a known species in the GTDB database are considered novel species, based on criteria including an ANI of less than 0.95 and an alignment fraction exceeding 0.5. To construct the phylogenetic tree of all representative MAGs, a maximum-likelihood phylogenetic tree was built using PhyloPhlAn (version 3.0.2) [12] based on the assembled genome sequences. This tree was subsequently visualized using the Interactive Tree of Life (iTOL, version 6.5.2) [13].

We have developed an online database named “Pbac” to store these MAGs, offering free search and download services. This database was constructed using the Django framework and Python, and it has been deployed on Alibaba Cloud. Detailed descriptions of the additional services provided by this database can be found on the website (<http://www.pbac.top>).

**2. ARG and MGE identification**

The representative MAGs were subjected to gene prediction using Prodigal software [14]. ARGs identification was achieved by using the DeepARG v2 software (LS model) [15] with the parameters of "--min-prob 0.8 --arg-alignment-identity 50 --arg-alignment-evalue 1e-10 --arg-num-alignments-per-entry 1000". The mobile genetic elements (MGEs) were identified by aligning protein sequences of genes against the MGE Database “MobileGeneticElementDatabase” created by Parnanen et al. [16] using DIAMOND (version 2.0.12) [17] with the criteria of e-value ≤ 10^-5^, > 80% sequence identity, and > 80% query coverage. We identified the occurrence of MGEs and ARGs using a custom Python script designed for data processing.

The human microbial genome information was retrieved from the NCBI/Genome database, accessed on September 4, 2023. The selection was based on filtering by host, specifically for 'human' or 'Homo sapiens' entries. Then, we randomly selected from these genomes and downloaded microbial genomes from the NCBI/Genome database. After removing shorter genomes ( < 200 kb), a total number of 13,042 microbial genomes were used for downstream analysis. The Prodigal (v2.6.3) [14] was used for gene prediction of the selected human-related genomes. The ARGs were identified using DeepARG v2.0 software (LS model) with the parameters of “--min-prob 0.8 --arg-alignment-identity 50 --arg-alignment-evalue 1e-10 --arg-num-alignments-per-entry 1000”. The giant panda-related ARGs were clustered with 99% similarity using CD-hit-est software (version 4.8.1) [18], and then mapped to human-related ARG sequences to identify the potential homologous ARG genes using CD-hit-est-2d (version 4.8.1) [18] with 99% similarity [19].

**3. Abundance, Expression, and Differential Analysis**

The raw meta-transcriptomic data from the giant panda microbiotas underwent quality control, host-contaminant removal, and rRNA filtering, as detailed in our previous study [1]. The abundance of ARG reads in various samples, including both metagenomic and meta-transcriptomic samples, was calculated using Salmon software (v1.6.0) with quant mode [20]. This involved mapping the metagenomic and meta-transcriptomic reads to ARG gene sequences. To calculate the relative abundance and expression of ARGs across different samples, the abundance of ARG genes was normalized to a count per million reads. We employed DESeq2 [21] to investigate the variations in ARGs between wild and captive populations of giant pandas, applying a significance threshold of adjusted *p*-value < 0.05 and a fold change (|FC|) greater than 1. In this analysis, only genes that were represented by a minimum of five reads in at least ten samples were considered.

**Reference**

1. Deng, Feilong, Chengdong Wang, Desheng Li, Yunjuan Peng, Linhua Deng, Yunxiang Zhao, Zhihao Zhang, et al. 2023. “The unique gut microbiome of giant pandas involved in protein metabolism contributes to the host’s dietary adaption to bamboo.” *Microbiome* 11: 180. <https://doi.org/10.1186/s40168-023-01603-0>

2. Guo, Wei, Sudhanshu Mishra, Chengdong Wang, Hemin Zhang, Ruihong Ning, Fanli Kong, Bo Zeng, Jiangchao Zhao, Ying Li. 2019. “Comparative study of gut microbiota in wild and captive giant pandas (Ailuropoda melanoleuca).” *Genes* 10: 827. <https://doi.org/10.3390/genes10100827>

3. Bolger, Anthony M, Marc Lohse, Bjoern Usadel. 2014. “Trimmomatic: a flexible trimmer for Illumina sequence data.” *Bioinformatics* 30: 2114-2120. <https://doi.org/10.1093/bioinformatics/btu170>

4. Langmead, Ben, Steven L Salzberg. 2012. “Fast gapped-read alignment with Bowtie 2.” *Nature Methods* 9: 357-359. <https://doi.org/10.1038/nmeth.1923>

5. Antipov, Dmitry, Anton Korobeynikov, Jeffrey S McLean, Pavel A Pevzner. 2016. “hybridSPAdes: an algorithm for hybrid assembly of short and long reads.” *Bioinformatics* 32: 1009-1015. <https://doi.org/10.1093/bioinformatics/btv688>

6. Olm, Matthew R, Christopher T Brown, Brandon Brooks, Jillian F Banfield. 2017. “dRep: a tool for fast and accurate genomic comparisons that enables improved genome recovery from metagenomes through de-replication.” *The ISME journal* 11: 2864-2868. <https://doi.org/10.1038/ismej.2017.126>

7. Kang, Dongwan D, Feng Li, Edward Kirton, Ashleigh Thomas, Rob Egan, Hong An, Zhong Wang. 2019. “MetaBAT 2: an adaptive binning algorithm for robust and efficient genome reconstruction from metagenome assemblies.” *PeerJ* 7: e7359. <https://doi.org/10.7717/peerj.7359>

8. Parks, Donovan H, Michael Imelfort, Connor T Skennerton, Philip Hugenholtz, Gene W Tyson. 2015. “CheckM: assessing the quality of microbial genomes recovered from isolates, single cells, and metagenomes.” *Genome research* 25: 1043-1055. <https://doi.org/10.1101/gr.186072.114>

9. Chan, Patricia P, Brian Y Lin, Allysia J Mak, Todd M Lowe. 2021. “tRNAscan-SE 2.0: improved detection and functional classification of transfer RNA genes.” *Nucleic acids research* 49: 9077-9096. <https://doi.org/10.1093/nar/gkab688>

10. Chaumeil, Pierre-Alain, Aaron J Mussig, Philip Hugenholtz, Donovan H Parks. 2019. “GTDB-Tk: a toolkit to classify genomes with the Genome Taxonomy Database.” *Bioinformatics* 36: 1925-1927. <https://doi.org/10.1093/bioinformatics/btz848>

11. Parks, Donovan H, Maria Chuvochina, Christian Rinke, Aaron J Mussig, Pierre-Alain Chaumeil, Philip Hugenholtz. 2022. “GTDB: an ongoing census of bacterial and archaeal diversity through a phylogenetically consistent, rank normalized and complete genome-based taxonomy.” *Nucleic acids research* 50: D785-D794. <https://doi.org/10.1093/nar/gkab776>

12. Asnicar, Francesco, Andrew Maltez Thomas, Francesco Beghini, Claudia Mengoni, Serena Manara, Paolo Manghi, Qiyun Zhu, Mattia Bolzan, Fabio Cumbo, Uyen May. 2020. “Precise phylogenetic analysis of microbial isolates and genomes from metagenomes using PhyloPhlAn 3.0.” *Nature communications* 11: 2500. <https://doi.org/10.1038/s41467-020-16366-7>

13. Letunic, Ivica, Peer Bork. 2019. “Interactive Tree Of Life (iTOL) v4: recent updates and new developments.” *Nucleic acids research* 47: W256-W259. <https://doi.org/10.1093/nar/gkz239>

14. Hyatt, Doug, Gwo-Liang Chen, Philip F LoCascio, Miriam L Land, Frank W Larimer, Loren J Hauser. 2010. “Prodigal: prokaryotic gene recognition and translation initiation site identification.” *BMC bioinformatics* 11: 1-11. <https://doi.org/10.1186/1471-2105-11-119>

15. Arango-Argoty, Gustavo, Emily Garner, Amy Pruden, Lenwood S Heath, Peter Vikesland, Liqing Zhang. 2018. “DeepARG: a deep learning approach for predicting antibiotic resistance genes from metagenomic data.” *Microbiome* 6: 1-15. <https://doi.org/s40168-018-0401-z>

16. Pärnänen, Katariina, Antti Karkman, Jenni Hultman, Christina Lyra, Johan Bengtsson-Palme, DG Joakim Larsson, Samuli Rautava, et al. 2018. “Maternal gut and breast milk microbiota affect infant gut antibiotic resistome and mobile genetic elements.” *Nature communications* 9: 3891. <https://doi.org/10.1038/s41467-018-06393-w>

17. Buchfink, Benjamin, Chao Xie, Daniel H Huson. 2015. “Fast and sensitive protein alignment using DIAMOND.” *Nature Methods* 12: 59-60. <https://doi.org/10.1038/nmeth.3176>

18. Li, Weizhong, Adam Godzik. 2006. “Cd-hit: a fast program for clustering and comparing large sets of protein or nucleotide sequences.” *Bioinformatics* 22: 1658-1659. <https://doi.org/10.1093/bioinformatics/btl158>

19. Hu, Yongfei, Xi Yang, Jing Li, Na Lv, Fei Liu, Jun Wu, Ivan YC Lin, et al. 2016. “The bacterial mobile resistome transfer network connecting the animal and human microbiomes.” *Applied and Environmental Microbiology* 82: 6672-6681. <https://doi.org/10.1128/AEM.01802-16>

20. Patro, Rob, Geet Duggal, Michael I Love, Rafael A Irizarry, Carl Kingsford. 2017. “Salmon provides fast and bias-aware quantification of transcript expression.” *Nature Methods* 14: 417-419. <https://doi.org/10.1038/nmeth.4197>

21. Love, Michael I, Wolfgang Huber, Simon Anders. 2014. “Moderated estimation of fold change and dispersion for RNA-seq data with DESeq2.” *Genome biology* 15: 1-21. <https://doi.org/10.1186/s13059-014-0550-8>


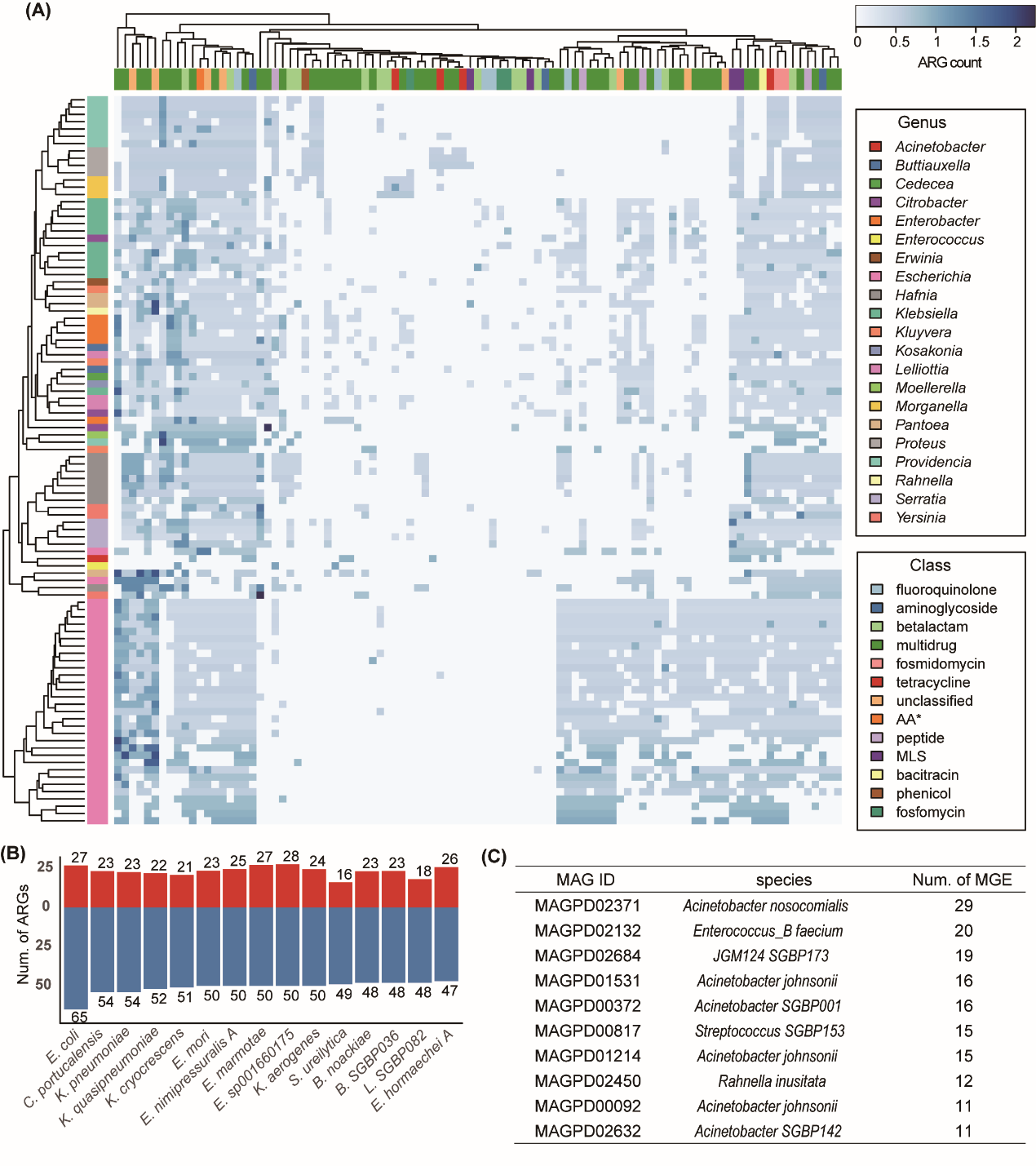
Figure S1. Predominant hosts of ARGs and MGEs at the species and strain levels. (A) A heatmap illustrating the distribution of antibiotic resistance genes (ARGs) among the top abundant bacterial species that carry the highest number of ARGs. The x-axis denotes the various types of ARGs, while the y-axis represents the bacterial species. (B) The mean count of ARGs per species is presented. The upper value denotes the ARGs density, represented as average counts per two thousand genes. In contrast, the lower value indicates the number of distinct ARG types identified within each species. (C) Tabulation of the top 10 MAGs acting as carriers for mobile genetic elements (MGEs).
